# Big Biology: Supersizing Science During the Emergence of the 21st Century

**DOI:** 10.1007/s00048-016-0141-8

**Published:** 2016-06

**Authors:** Niki Vermeulen

**Affiliations:** Science, Technology and Innovation Studies, School of Social and Political Science, University of Edinburgh, Chisholm House, High School Yards, Edinburgh, EH1 1LZ, UK

**Keywords:** Großforschung, Zusammenarbeit, Innovation, Lebenswissenschaften, Systembiologie, Large-scale research, collaboration, innovation, life sciences, systems biology

## Abstract

Ist Biologie das jüngste Mitglied in der Familie von Big Science? Die vermehrte Zusammenarbeit in der biologischen Forschung wurde in der Folge des *Human Genome Project* zwar zum Gegenstand hitziger Diskussionen, aber Debatten und Reflexionen blieben meist im Polemischen verhaftet und zeigten eine begrenzte Wertschätzung für die Vielfalt und Erklärungskraft des Konzepts von Big Science. Zur gleichen Zeit haben Wissenschafts- und Technikforscher/innen in ihren Beschreibungen des Wandels der Forschungslandschaft die Verwendung des Begriffs Big Science gemieden. Dieser interdisziplinäre Artikel kombiniert eine begriffliche Analyse des Konzepts von Big Science mit unterschiedlichen Daten und Ideen aus einer Multimethodenuntersuchung mehrerer großer Forschungsprojekte in der Biologie. Ziel ist es, ein empirisch fundiertes, nuanciertes und analytisch nützliches Verständnis von *Big Biology* zu entwickeln und die normativen Debatten mit ihren einfachen Dichotomien und rhetorischen Positionen hinter sich zu lassen. Zwar kann das Konzept von Big Science als eine Mode in der Wissenschaftspolitik gesehen werden – inzwischen vielleicht sogar als ein altmodisches Konzept –, doch lautet meine innovative Argumentation, dass dessen analytische Verwendung unsere Aufmerksamkeit auf die Ausweitung der Zusammenarbeit in den Biowissenschaften lenkt. Die Analyse von *Big Biology* zeigt Unterschiede zu *Big Physics* und anderen Formen von Big Science, namentlich in den Mustern der Forschungsorganisation, der verwendeten Technologien und der gesellschaftlichen Zusammenhänge, in denen sie tätig ist. So können Reflexionen über Big Science, *Big Biology* und ihre Beziehungen zur Wissensproduktion die jüngsten Behauptungen über grundlegende Veränderungen in der Life Science-Forschung in einen historischen Kontext stellen.

Discussing the Human Genome Project, a 2001 *Nature* editorial concluded: “Like it or not, big biology is here to stay” (2001: 545). This statement aimed to make a final conclusion in heated debates on big biology that started during the emergence of the Human Genome Project. In these discussions, the project was portrayed as the first big science project in biology (Kevles and Hood 1992) and as the Manhattan Project of biology (Lenoir and Hays 2000), thereby framing transformations in the life sciences in terms of growth and comparing it to the emergence of big physics. Proponents of big biology presented the Human Genome Project as the new and more effective way to perform research, while opponents claimed it undermined the very character of biological research, bureaucratizing and politicizing it while diluting creativity (Check and Castellani 2004; Davis 1990). Genome sequencing was portrayed as “massive, goal-driven and mind-numbingly dull” (Roberts 2001). Molecular biologist Sydney Brenner even joked that sequencing was so boring it should be done by prisoners: “the more heinous the crime, the bigger the chromosome they would have to decipher” (Roberts 2001: 1183). Moreover, the term big biology was mainly used as a rhetorical weapon in the debate between scientists and policymakers in favor of the Human Genome Project and those opposing large-scale projects in biology. While the big science concept has a long history and acquired rich and multi-faceted meanings over time, debates about big biology focused on overly simple framings and uncritical conceptualizations. Moreover, big science was pictured as either good or bad, prematurely silencing the development of more nuanced understandings of the positive and negative sides of ongoing transformations in the biosciences.

The decoupling between debates about big biology and the meanings of the concept of big science can be explained in part by the fact that these debates have been mainly conducted by life scientists rather than scholars of science and technology studies. The latter have been hesitant to use the big science concept and prefer other perspectives for studying the Human Genome Project and related transformations in biological research, focusing on administration, standardization, informatisation, identity formation, ethics and politics (Balmer 1996; Calvert 2013; Cook-Deegan 1994; Hilgartner 1995; Garcia-Sancho 2012; Glasner 1996; Glasner 2002; Sloan et al. 2000).^1^ Thereby the most important criticism of the concept concerns its broadness, with some arguing that it is too broad, vague, and ambiguous to be analytically useful for analyzing contemporary transformations of scientific research and practice (Rechsteiner 1990; Shrum et al. 2007).

In contrast, I innovatively argue that the broadness of the big science concept can also be its strength, provided that its different meanings are acknowledged and distinguished. Not using the empirical understanding of the concept in analysis of contemporary large-scale biology, means cutting loose from important historical observations on the growth of science and the increase of collaboration that can give important insights in the specificity of contemporary developments. The concept connects the growth of biology to similar developments in other scientific fields, most notably physics research, and places them in a historic and cultural context. Moreover, the big science concept adds to more recent (historical) conceptualisations of transformations in science—such as Mode 2 science, normal and post-normal science, and the triple helix theory (Gibbons et al. 1994; Etzkowitz and Leydesdorff 2000; Funtowicz and Ravetz 1993, 1994; Nowotny et al. 2001)—as it explicitly addresses issues of scale and the increase of scientific collaboration in combination with attention for the transforming context in which science takes place.

As such, this interdisciplinary paper first presents the multi-faceted character of the big science concept, after which it explores how big science enables the understanding of transformations in biology. By combining historical and sociological studies, I outline the growth of biology through an empirical understanding of the concept, asking how big biology differs from big physics, explicitly paying attention to the interaction between the epistemic and organizational. Focusing first on technology as a driver for growth and second on the societal context which enables this scientific growth, I argue that in contrast to the centralized form of big physics, big biology has acquired a networked shape in interaction with the central role that information technologies play in bioscience research. In addition, ideas about the role of science and technology in society have changed substantively since physics grew big, resulting in some specific obstacles that life scientists encounter in their efforts to scale-up biology. For instance, and although current science policy favours research that combines focus and mass with clear societal implications, life scientists find it difficult to acquire funding for large-scale biology while presenting claims to improve human health and the environment. This is especially remarkable when compared to particle physics or space research that, with less straightforward societal effects, is continuously able to acquire substantial amounts of funding. Or as one of the life scientists puts it: “Even when these space shuttles explode, they still receive huge amounts of money and we are not able to acquire this kind of funding for our research”.^2^ The final part of this chapter will argue, that these and similar obstacles around internationalization and innovation can be better understood when looking at biology through the lens of big science, showing how interactions between scientific and contextual developments shape the process of making science big (Capshew and Rader 1992), or what I have called the “supersizing of science”.

My argument is based on a mix of philosophical, historical and sociological research methods. It combines a conceptual analysis of the concept of big science, with explorations into the history of collaboration in biology and its present forms. For the analysis of the latter, I used different data and ideas from a multi-method investigation of several large-scale research projects in biology, covering ecology, molecular biology and biomedical science. I have especially concentrated on the emergence, structure and functioning of various large-scale academic projects in the United States and Europe (Parker et al. 2010; Vermeulen 2009; Vermeulen et al. 2010; Vermeulen 2012; Vermeulen et al. 2013).^3^ Studies involved document analysis, interviews with scientists and policymakers as well as participant observations.

## The Meanings of Big Science

Opening the black-box of big science forms a starting point for exploring the way in which the concept can help to grasp contemporary large-scale biology research. The term was coined in 1961 by physicist Alvin Weinberg in the context of the enlargement of physics research in the United States (Weinberg 1961) and further developed by historian of science Derek de Solla Price in his book *Little Science, Big Science* (1963): “The large-scale character of modern science, new and shining and all powerful, is so apparent that the happy term “Big Science” has been coined to describe it” (ibid.: 2). In his book *Reflections on Big Science* (1967) Weinberg states that science has become big in two different ways:

On the one hand, many of the activities of modern science—nuclear physics, or elementary particle physics, or space research—require extremely elaborate equipment and staffs of large teams of professionals; on the other hand, the scientific enterprise, both Little Science and Big Science, has grown explosively and has become very much more complicated (ibid.: 39).

The big science books are part of a long list of books that all address growth as a distinctive phenomenon of modern society, like *Big Business* (Fay 1912; Hendrick 1919; Drucker 1947), *Big Government* (Pusey 1945), *Big Democracy* (Appleby 1945), *Big School* (Barker 1964), *Big Cities* (Rogers 1971), *Big Foundations* (Nielsen 1972) and *Big Machine* (Jungk 1986). Like the other authors of these big books, Weinberg and De Solla Price write about increasing dimensions full of wonder and admiration, while at the same time evaluating them critically. Growth is described as part of progress and an inevitable exponent of modern industrial society, but it is also seen as a source of problems. For instance, Peter Drucker (1947) talks about the “Curse of Bigness” (ibid.: 211) and economist Schumacher wrote a book entitled *Small is Beautiful* (1973). Thereby the books on bigness breathe the ambivalence of modern condition: “To be modern is to find ourselves in an environment that promises us adventure, power, joy, growth, transformation of ourselves and the world— and, at the same time, that threatens to destroy everything we have, everything we know, everything we are” (Berman 1983: 15). Accordingly, from its emergence the concept of big science has an ambivalent understanding of growth that is characteristic for the modern condition and which is still very much visible in the two opposing views on big science in the debate on big biology.

Such ambivalent views are also reflected in the first writings on big science by Weinberg and De Solla Price. Weinberg’s attention to growth in science is the product of his participation in America’s large nuclear energy projects as director of the Oak Ridge National Laboratory (ORNL) (Schaffer 1998; Weinberg 1994).^4^ Accordingly, *Reflections on Big Science* (Weinberg 1967) primarily argues that large-mission oriented laboratories are indispensable to society. However, it also attempts to come to terms with the most troubling questions: the allocation of resources among competitive fields, between science and other public enterprises, and whether “Big Science is blunting science as an instrument for uncovering new knowledge” (Weinberg 1967: vi). And while Price seems primarily impressed by the growth of science, and the transformation from small to big, he also observes that the extrapolation of these growth rates in science would soon show a world populated by only scientists, which would be worrying indeed (Price 1963).

In addition, De Solla Price introduces big science as an empirical concept for studying transformation in science. Originally a physicist, he became interested in the history of science when he received a complete set of the *Philosophical Transactions of the Royal Society of London* from 1662 to the 1930s, while teaching in Singapore (Price 1983). The pile of journals triggered his fascination for what he later would call big science.

I took the beautiful calf-bound volumes into protective custody and set them in ten-year piles on the bedside bookshelves. For a year then I read them cover to cover, thereby getting my initial education as a historian of science. As a side product, noting that the piles made a fine exponential curve against the wall, I counted all the other sets of journals I could find and discovered that exponential growth, at an amazingly fast rate, was apparently universal and remarkably long-lived (ibid.: 18).

This discovery of exponential growth stimulated his work on the quantitative measurement of scientific development that turned him into a recognized information scientist and a founding father of scientometrics (Crawford 1984; Garfield 1984; Mackay 1984). De Solla Price introduced big science primarily as an empirical concept, pushing the limits of the history of science from studying the past to studying the present while also giving it a quantitative turn.

However, next to being a quantitative empirical phenomenon, the concept of big science became also used for the qualitative study of scientific transformation. Against the background of the development of the history and social study of science and technology, big science has been employed to look into historic and contemporary practices of research collaboration: “Where past studies on big science typically counted dollars and personnel, and tabulated the funding sources that nourished large-scale research, we can now see more of the causes and consequences of the growth of science” (Hevly 1992: 357). Detailed case studies of different forms of big science in fields as diverse as astronomy, ecology, physics, and space research enriched empirical understanding the phenomena (Bocking 1997; Crease 1999; Galison and Hevly 1992; Galison 1997; Hoddeson and Baym 1993; Kwa 1987; Lambright 1998; Schloegel and Rader 2005; Westwick 2003). Common features are found in large, expensive instruments, industrialization, centralization, multi-disciplinary collaboration, institutionalization, science-government relations, cooperation with industry and internationalization. Moreover, the emergence of large-scale research complexes is perceived as a broader trend, and while different types of big science have their own characteristics as differences in discipline and time account for variety, they also display family resemblances (Wittgenstein 1953).

In sum, big science is an historic concept formed in the 1960s to reflect on increasing scales and dimensions of research and scientific practice, and the term has taken on a variety of meanings and functions. Consequently, it now has both empirical and evaluative aspects, each with a double-edge. Empirical investigations can be divided into quantitative and qualitative approaches, while both positive and negative evaluations of the phenomenon of big science are distinguishable. As such, and when thinking about what big biology would entail, it is important to keep these different meanings of big science in mind. The debate about the Human Genome Project that questioned the desirability of the rise of big biology, clearly draws on the evaluative side of the big science concept, while failing to unpack what big biology actually entails in practice. How does big biology look, and how do increasing dimensions in biology research transform the character of this research? Such reflections on the process of biology becoming big science will need to use the empirical side of the concept, as the next section will demonstrate.

## Exploring the Character of Big Biology

Gaining a full empirical appreciation of big biology requires understanding its origins, development, and how it compares to large-scale scientific research in other fields.^5^ Big physics will function as reference point for established big science, as this is the exemplary situation that formed the big science concept. In addition, a direct connection exists between big physics and contemporary collaboration in biology. For instance, the United States Department of Energy that is known for organizing big physics research also played an important role in setting-up the Human Genome Project.^6^ However, how does big biology differ from the well-known collaborations in physics? This section shows how big biology combines a specific history with different organizational developments around technology, while it also emerged in a particular social context. After outlining the history of big biology and detailing specific organizational movements towards aggregation, centralization and networking, I will focus on the different societal embeddings of big physics and big biology. While physics grew in the beginning of the twentieth century, large-scale biology only became prominent towards the end of the twentieth century. As these two periods are quite different in terms of international relations and social order as well as regarding the place of science in society, I will explore how these differences have influenced the specific forms of big science.

However, before exploring the history and current status of biology, it is important to emphasize that not all of academic biology research is becoming big, and this paper should certainly not be read as a promotion of bigness. Instead, I acknowledge that different forms of research into life require different organisational scales (Vermeulen et al. 2010), and as such this paper explicitly discusses specific branches of biology that are building larger-scale collaborations, most notably ecology, molecular biology and biomedical science.

### A History of Big Biology

*Reflections on Big Science* (Weinberg 1967) already predicted biology becoming the next big science:

We are, or ought to be, entering an age of biomedical science and biomedical technology that could rival in magnitude and richness the present age of physical science and physical technology. Whether we shall indeed enter this age will depend upon the attitude toward Big Biology adopted by biomedical scientists and government agencies that support biology (ibid: 101).

Weinberg’s thoughts on big biology were inspired by Norman Anderson, a “biologist-cum-engineer” who in 1967 proposed the Molecular Anatomy program, aiming to catalogue and characterize all human proteins (Weinberg 1999: 738). Nevertheless, the Human Proteome Organisation was not launched until 2001—fostering international initiatives to investigate human proteins, the field currently known as, proteomics’—and was thus preceded by the Human Genome Project.

However, and in line with the dominance of physics research during most of the twentieth century, the predominant focus of historical and sociological analyses of big science have been on physics and the emergence of its large-scale instrumentation. The origin of big physics was traced back to the interwar period when universities in California began collaborating to find a solution for the problems of power production and distribution (Galison 1992; Seidel 1992). Large-scale physics research spread internationally after the important contribution of large-scale physics research to World War II. Contemporary research focuses primarily on particle physics, for example at the European Organization for Nuclear Research (Knorr 1999; Galison 1997). But what about big biology?

With physics commonly known as big science, biology is only recently portrayed as such, with the Human Genome Project functioning as a watershed. Too often ignored is the fact that large-scale physics research gave rise to radiobiology well before the current era, and that biologists collaborated in large-scale research enterprises long before large-scale genomics and post-genomics research (Creager and Santesmases 2006; Lenoir and Hays 2000; Schloegel and Rader 2005; Westwick 2003). More specifically, the history of ecology describes some earlier large-scale endeavors to collect, catalogue and analyze research material with a dispersed character (Aronova et al. 2010; Bocking 1997; Kwa 1987). In addition, increases in the scale of the biosciences occurred already in interaction with the emergence of molecular biology in the late 1950s and 1960s (De Chadarevian 2002; Magner 1994; Strasser 2003a, b; Rose 2001). In Europe the creation of CERN in 1953 and the establishment of the European Space Research Organisation (ESRO) in 1962 were followed by the creation of the European Molecular Biology Organization (EMBO) and the European Molecular Biology Laboratory (EMBL) in 1964 and 1974 respectively. At that time organizations such as the Council of Europe, EURATOM, NATO, OECD, UNESCO and the WHO were inciting international collaborations in the life sciences and the creation of international laboratories: “We tend to forget that in the 1960s, EMBO had to face many competing projects to develop international cooperation in the life sciences at the European, Atlantic and global level.” (Strasser 2003a: 542).

This international institutionalization mirrored the centralization of research in particle physics, but it was followed by international mapping and sequencing projects that scaled-up research efforts through collaboration but without centralization in one place (Garcia-Sancho 2012; Gaudilliére and Rheinberger 2004; Kohler 1994; Sulston and Ferry 2002). Collaborations based around model systems such as Drosophila and C. Elegans had a networked character and were particularly important as forerunners of the Human Genome Project. Built around fly and worm, connections were formed by scientists to exchange research material and information as well as to divide labor across laboratories. This in turn set the stage for the collaborative work on the human genome and the international Human Genome Project.

Although perspectives on the Human Genome Project are diverse, all agree that the project has become an exemplar of new scientific, technological, and organizational transformations (Cantor 1990; Kevles and Hood 1992; Kevles 1997; Glasner 2002; Hillgartner 1995). The magnitude and complexity of the project is impressive. The public consortium consisted of 16 research groups spread across 48 laboratories world-wide with additional efforts in the private realm; the publication of the completed human genome sequence named 520 scientific authors (Glasner 2002). The Human Genome Project is considered the foundation of major current research endeavors in the, post-genomics era,’ including new integrative approaches in the biosciences (Fujimura 2005; Webster 2005). As such, it is perceived as a major turning point initiating new styles of biological research.

### New Socio-technical Arrangements in Biology

With the emergence of many large-scale research collaborations in biology it seems to have become legitimate to talk about big biology. Nevertheless, I argue that important differences can be noted between big physics and big biology. Most importantly, reasons for collaboration in biology have initially been found in the dispersed character of the research material instead of the increasing size of instruments. Large and expensive instruments are often seen as essential to the emergence of big science, as in big physics particle accelerators cause organizational gravitation (Capshew and Rader 1992). Large instruments are the most important incentive to centralize because they cannot be built and maintained by any one lab, university, or country. However, instruments in biology are relatively small, and it is often argued that biology is not big science as technologies do not come close to the size of instruments such as particle accelerators in high-energy physics. So the question arises: what socio-technical arrangements characterize big biology research? By examining interactions between technological developments and organizational changes in biological research I distinguish three different movements: aggregation, centralization and networking. These processes are respectively connected to the introduction of larger research instruments, the creation of technology centers, and the growing importance of information and communication technologies, which makes life scientists and their research projects part of an international network of data and databases.

First of all, biologists are employing larger research instruments, leading to higher levels of aggregation in the organization of biological research that resemble centralization around large instruments in physics, albeit on a smaller scale. To illustrate, the introduction of the Nuclear Magnetic Resonance Spectroscope^7^ in biology exemplifies the emergence of aggregation in biology research. Although NMR spectroscopy has its origins in the physical and chemical sciences it became employed in biology to study molecular structures from the 1930s onwards (Zallen 1992). The integration of the NMR spectroscope in biological research was part of a broader effort of the Rockefeller Foundation to stimulate cross-disciplinary research, and it stimulated the further development of the instrument. At the same time it increased the costs^8^ and the scales of research arrangements substantively, constructing national and international collaborations.

Dr. Rien de Bie—who spent a considerable amount of his career as research manager in the Bijvoet Centre for Biomolecular Research at Utrecht University in the Netherlands—witnessed these increasing scales of research arrangements around the instrument firsthand.^9^ De Bie recalled how the first NMR spectrometer devoted to biological research entered Utrecht University in 1963. While initially only used locally, in the 1980s a first move towards larger scales occurred when a shift from local ad-hoc financing of research instruments to structural investments in a national context took place:

It was during this period that in the Netherlands the idea developed that if you want to use expensive technologies—which means electron-microscopy, NMR spectroscopy, röntgendefraction, ultracentrifuges and that kind of things—you have to start thinking seriously about how to go along.^10^

Based on existing practices in physics, this resulted in a policy of concentration and the establishment of so-called, para-university research institutes’ for chemistry and molecular biology. Consequently, in 1988 three national collaborative centers were funded, including the Bijvoet Center in Utrecht. The next increase of scale was mainly policy-induced, as internationalization became a priority in research policy from the end of the 1980s onwards. As in the first European Union Framework Programmes no research infrastructures were funded on the biological side of chemistry, Utrecht decided to participate together with centers in Florence and Frankfurt in the third Framework Programme to fill this void, leading to the establishment of European collaborations for NMR spectroscopy that exist to this day. De Bie characterized this final step as the, Europeanisation’ of research arrangements.

Next to the (inter)national aggregation of efforts around one single technology, a second organizational movement in biology consists of local centralization of people and ideas around several different technologies, as recognized in the establishment of local central technology facilities. Researchers and laboratories traditionally bought their own research technologies, but this strategy now limits the kinds of research to be conducted (Perkel 2006).^11^ It is increasingly difficult to buy all the technologies needed when starting a lab. In addition, keeping technology up-to-date and functional requires substantial resources. This has given rise to practices such as sharing or leasing laboratory equipment, the use of so-called, kits’ or black-boxed tests that perform already standardized research processes, and outsourcing to (commercial) service centers (Kleinman 2003; Wolthuis 2006). More importantly in this context, in-house technology centers have been developed, places in universities or research institutes where scientists go to make use of specific technologies.

The Technology Facility in the Biology Department at the University of York in the United Kingdom is an early example of this trend towards centralization around the technological repertoire in biology.^12^ Professor Dianna Bowles, who led the development of the Technology Facility said that they wanted it to function as a hub within the department to provide scientists with advanced technologies:

You can be interested in spiders or in plants or whatever, and they are all underpinned by the same technology platform. But individual research groups do not need to use those technologies 100% of their time, they just jump in and out when their research dictated the need. So the best way to do it, is actually to put all of these technologies in one place.^13^

Such facilities give researchers access to otherwise unavailable technologies, shaping the character of their research and keeping them competitive with industrial research efforts. The central idea behind this center is not only the sharing of costs, but also the development of professional operational skills. With the growing complexity of instruments, the knowledge and skills required in using them increases, and operational expertise becomes evermore crucial. Nowadays, it is often a full-time job to keep up with the latest technology developments, thus requiring specialists. Therefore the Technology Facility in York does not only concentrate technologies, but expertise and training as well. Moreover, the center propels new collaborations as scientists with different research aims, skills and backgrounds get together through their need to use the same techniques, sharing their experiences and expertise in the process.^14^

The third movement does not aggregate or concentrate research in biology but links researchers into networks. Building on the exchange of research material, and in interaction with the integration of information technologies and the informatisation of biology research (Beaulieu 2004; Keller 1995; Kay 2000), a networked form of research collaboration emerged. Based on ideas of biology as information, sequencing methods emerged that developed within networks around flies, worms and humans (Kohler 1994; Garcia-Sancho 2012; Gaudilliére and Rheinberger 2004; Sulston and Ferry 2002). These sequencing projects produced enormous amounts of data, making storing and integrating data a key feature of modern biology (Groenewegen and Wouters 2004; Leonelli 2014). A variety of data is brought together by building (online) databases that are dispersed all over the world, but can be accessed from multiple locations.^15^ Research collaboration is thus not performed in one single location, but the places of research are dispersed yet connected through information infrastructures. As such, the integration of information technologies in biology facilitated the building of connections between relatively small and dispersed sites of knowledge production, giving collaboration in biology a networked character (see also Glasner 1996).

In sum, the three organizational movements surrounding technology in biology resemble only partially the organizational configurations in big physics. On the one hand, (inter)national aggregation around larger instruments and local centralization around complex and expensive instruments resemble centralization in physics, although on a smaller scale. On the other hand the networked character sets big biology apart. Although in large-scale physics research the analysis of data now takes place in international dispersed networks as well, these network structures are still built around centralized experiments for data generation which makes it different from the network structures in biology in which both data production and analysis is dispersed.^16^ Moreover, this movement is not a feature unique to biology given that it reflects socio-technical transformations around information technologies in science and society at large (Abbate 1999; Van Dijk 1991; Castells 1996; Groenewegen and Wouters 2004; Hine 2006), and as such technological transformations in biology must also be considered within these societal contexts.

### The Social Context of Big Biology

From the various organizational movements in biology it becomes apparent that not only technological developments have influenced the growth of research. Funding organizations have played a role in shaping connections between different disciplines and provided for the development of new ways to organize research, locally and in an international context. The political movements towards national concentration of research on the one hand, and the creation of the European Framework Programmes and the European Research Area on the other hand, have contributed to increasing scales in life sciences research. In addition, competition and cooperation with industry influenced academic research arrangements. Thereby, individuals play an important role, for instance in reshaping the local organization of research in line with international developments, connecting local, national and international levels. However, not only the internal dynamics of science shape research arrangements but also external factors influence big science complexes, and big biology can only be understood in the light of the social environment in which it emerged.

While big physics emerged in the early twentieth century and was conceptualized in the 1960s, it was not until the end of the twentieth century that large-scale research arrangements in biology became prominent. These two periods are quite different in terms of international relations and the ordering of societies, and also with respect to the place of science in society, influencing specific forms of big science. After discussing the societal context in which big physics emerged, I will show how in the second half of the twentieth century a shift can be observed towards research into life, which grew big in quite a different societal context. Differences discussed will include the context of emergence, and focus on relations to government industry and society, as visible in Table 1.

Starting with a focus on the context in which big physics emerged, we have to look at the interbellum in the United States as well as the subsequent World War II and its aftermath. More specifically, Galison (1992) points out that the roots of big physics are in the Great Depression in the United States, which caused a counter reaction that admired, bigness’ inspiring the enormous structures of the Golden Gate Bridge, the Hoover Dam, and the Empire State Building: “Without the cultural fascination of Americans in general for the large, the goal of building ever larger scientific facilities might have remained peripheral to other concerns” (Galison 1992: 3). Moreover, growth became a central phenomenon of modern society and was accompanied by a strong belief in progress and by the ordering and (re-)structuring of society (Berman 1983; Kumar 1995; Latour 1993). Rationalization and industrialization processes gave rise to Taylorism and Fordism—efficiency and scaling—and were accompanied by bureaucratization. Exactly these features of modernization are the ones visible in big physics that emerged during this period and developed into the well-known large military-academic physics research complexes in World War II (Galison 1992; Seidel 1992; Hoddeson and Baym 1993).

Although the Manhattan Project is frequently named as reference point for the emergence of large-scale physics, it is important to note that not only its emergence can be traced back to an earlier time, but also that the term, big science’ was coined after the war. Therefore, big physics should not only be understood in the context of World War II but also in the context of American society in the 1960s. In this after-war period the project of modernization continued, while important transformations took place in the relationship between science and society. During wartime governmental investments in science increased enormously, but also strengthened the political grip on the direction of scientific research. The end of the war was therefore seized as an opportunity to renegotiate the relation between government and science (Galison 1992; Guston 2000; Jasanoff 2005; Rip 1998). The social contract for science based on the famous report of Vannevar Bush (1945 [1980]) can be read as a way to (re-)establish the divide between government and science after the war, safeguarding the continuity of government investment in science while also creating a protected space for science. Science was put forward as an investment that would eventually pay off, using successful scientific applications of the war as a symbol for the societal usefulness of basic science. Writings on big physics reflect this view on science; fundamental science and the application of science go hand in hand and do not exclude each other.

Interestingly, a clear connection exists between big physics and big biology. As investments in physics became controversial—first due to the devastating power of the Atomic Bomb and later because of the debate on Nuclear Energy—large-scale research in physics had to reposition itself, which also resulted into the incorporation of biology research. National physics laboratories in the United States started to include research into life in their strategy, as this brought less controversial societal benefits and also fitted increasing attention for environmental pollution. Consequently, the large-scale research organizations for physics gave a new impetus to biology research, which continues to the present day (Bocking 1997; Creager and Santesmases 2006; Doing 2004; Schloegel and Rader 2005). Moreover, in the context of investigating the effects of radiation on the human genetic make-up, it was the United States Department of Energy (DoE) that first envisioned large-scale research collaboration dedicated to molecular biology. The DoE used its experience in organizing large-scale physics research for shaping the Human Genome Project together with the National Institutes of Health, which paved the way for other large-scale research projects in the context of genomics and related research.

In the meantime, the position of science in society has also changed substantively, shaping the creation of large-scale biology. National governments have mostly shifted from centralized steering to the decentralisation of power with a neo-liberal approach in a globalized world order (Pellizzoni et al. 2012). The transformation from the modern industrial to the post-industrial society gave rise to the knowledge society—in which science and technology substitute industrialization (Bell 1973; Drucker 1969; De Wilde 2001). From the 1980s onwards science and technology have been put forward as a means towards economic growth, accompanied by an increasing attention for innovation processes and innovation policies (Irvine and Martin 1994; Jasanoff 2005; Remington 1988; Rip 1998). Consequently, the emphasis on processes of innovation and the (industrial) application of science are central in the relation between science and society during the period in which biology grows big. This emphasis on science for innovation is clearly reflected in developments in biology research, where relations to industry are promoted, and knowledge about life has become a commodity, giving rise to the bioeconomy (Rose 2001, 2007; Sunder Rajan 2006; Thackray 1998; Waldby and Mitchell 2006).

In addition, big biology research has been an important factor in the broader movement towards the embedding of science in society (Gibbons et al. 1996; Nowotny et al. 2001). Biology’s potential for the improvement of human health and the living environment legitimizes biology research, but also makes it more complicated as it touches on important societal issues that might give rise to controversies, such as genetically modified food or stem-cell research (Hansen 2010; Thompson 2013). Although big physics too has generated some serious controversies, in the context of a growing biology research the belief in science for progress has been greatly diminished, which is in part due to the very results of big physics. Moreover, the biosciences touch on life itself, having effects on health, food and our definitions of what life is (Tamminen and Vermeulen 2012). In an attempt to come to terms with the societal dimensions of science, big biology therefore was the first form of big science that addressed these issues in an integrated way. The Human Genome Project was accompanied by an extensive program on the ethical, legal and social implications of genetic research, which set the stage for many more research programs into the interaction between biology and society. Consequently, especially within the context of large-scale biological research, embedding science in society has become more important.

### Biology as a Specific Form of Big Science

When using the big science concept as a heuristic device to study qualitative transformations in the organization of research in its historical and cultural context, it becomes apparent that collaboration in biology does not exactly resemble big physics: biology is a different form of big science. Big biology is characterized by its networked structure and the integration of information technologies. In addition, the history of collaboration in biology and the role that technology plays in the formation of these collaborations, show that technology is not the only incentive to collaborate and therefore resonates critique on the technological deterministic character of big science literature (Capshew and Rader 1992; Westfall 2003). Next to technology, the dispersed character of the research material, the research approach and political movements towards international scientific collaboration are shaping big biology. Moreover, when looking into the interaction between technology and organizational transformations in biology, it becomes clear that technologies are part of a process in which research arrangements are actively reshaped by people, organizations and science policy. Finally, I have shown how big biology is very much a product of our contemporary society: reflecting social movements towards decentralization, networking, globalization and innovation, while also paying attention to ethical, legal and social implications.

## New Perspectives on the Dynamics of Large-Scale Biology Research

The value of the big science perspective on increasing scales in biology research is not only to improve the understanding of the phenomenon in historical and cultural contextualization, but also in contributing to the understanding of the dynamics of large-scale research endeavors. An analysis of the making of big biology provides interesting new insights in large-scale biology research that can help to understand its functioning (Capshew and Rader 1992; Vermeulen 2009). To illustrate, this section will give some fresh perspectives on problems that arise in big biology, focussing on three obstacles that become apparent in cases of building large-scale collaboration: the supersizing obstacle, the internationalization obstacle and the innovation obstacle.

### The Supersizing Obstacle

Although biology now knows some impressive large-scale projects, both in field and in laboratory biology, investigations into the emergence of these projects presents scientists that are struggling to make biology big (Vermeulen 2009). Most notably, the Human Genome Project required substantial amounts of lobbying before becoming reality, and although it paved the way for (post-)genomics research investments worldwide, such large-scale efforts in biology require the readjustment of funding structures—which is anything but a straightforward process. While resistance can be found within the academic community—with scientists being apprehensive regarding a shift in funding from small to large (see also Vermeulen et al. 2010)—some proposed projects simply do not succeed in finding funding that suits the aspired scale. For instance, in, systems biology’ scientists propose large-scale collaboration to model (parts of) the human body, but several plans at European level have not been realized so far (such as ESF Forward Look on systems biology that intended among other things to set up a European Organization for Systems Biology as well as the European flagship project IT for the Future of Medicine, ITFoM, that aimed to further develop modeling for medical applications).^17^ Scientists putting big efforts into the supersizing of biology are puzzled by this. Why is it so difficult to make biology big? A question that becomes even more poignant in view of the fact that traditional forms of big science, such as big physics and space research, are still able to raise funds even though the anticipated societal benefits are less clear. How can we understand that it is hard to make biology big, even though such projects bring clear societal benefits?

Reflecting on this issue with the understanding of the character of big biology from the analysis above, an explanation for this obstacle can be found in the historical advantage of traditional big science over biology as a new form of big science. History shows that traditional big science was build in a time when governments had a strong steering role and science was a source of political power. Large-scale projects in science had a clear political function, like for instance CERN that contributed towards building a united Europe after World War II (Pestre and Krige 1992). Within this post-war context, an organization like CERN was able to create a solid organizational machinery. Over time they acquired extensive lobbying experience, which assists them in continuously securing political support, funding, and hence sustainability. As a result, CERN still proves politically robust as it is impossible for individual countries to withdraw from the international organizational formation of CERN. So the continuous funding of traditional forms of big science is primarily an effect of their established bigness: once big science is build, it is difficult to break it down again (Lambright 1998). Or as a physicist working at CERN phrases it: “Nowadays we would not be able to build CERN”^18^. He thereby not only refers to the historical advantage of big physics versus big biology, but also suggests that in the current science policy environment, big physics would not be able to emerge (see also Kevles (1997) on the death of the Superconducting Super Collider). Against this background, it is not difficult to understand that making biology big proves to be a challenge. There are no historical claims to bigness, no investments in large instruments, no prior international political commitments that require a follow-through, nor much experience in organizing lobby-work for big biology.

In the concrete case of supersizing systems biology, it becomes apparent how complicated the building of large-scale biology in contemporary society actually is. Systems biology is an integrative approach that aims to synthesize information on different components of life into models that mimic cellular or organismal processes, organs or even whole living organisms, in order to understand, calculate and predict life (Calvert and Fujimura 2011). As the building of models of life requires multi-disciplinary collaboration, critical mass, and international standardization, scientists argue that the scale of collaborations should even exceed the Human Genome Project. As such, and in the process of establishing this new research approach, life scientists have lobbied both inside and outside the scientific community, convincing fellow scientists, politicians and policymakers, as well as industry, of its importance (Vermeulen 2012). However, and while the concept of systems biology has been quite successful, international large-scale projects have not yet materialized.

Initially, many scientists were skeptical of the aims and ambitions of systems biology (e.g. Plasterk 1993), but now the label figures in different types of research (Bock von Wülfingen 2009), is firmly on the science policy agenda, and written on the wall of several dedicated research institutes. Local institutes of systems biology have been established in Japan, the United States, and various European countries. Yet Germany is the only country that developed a large-scale national research project to model the human liver, and in other countries and internationally the research is fragmented.^19^ At European level, systems biology has become a theme from the 7th Framework Programme onwards, but although these projects provide opportunities for international coordination they do not have the same coherence as the Human Genome Project.

Reasons for this fragmentation of research can be attributed to the division between scientists and their approaches and a lack of (inter)national scientific coordination as well as to the fact that science policy mostly gets shaped at national level, resulting in local funding. While some scientists are content with the current fragmentation and relatively small-scale efforts, others continuously try to scale-up, searching for appropriate sources of (international) funding (e.g. the Neercanne Initiative that is explicitly aims to discuss the scaling-up of biology and current efforts to design a European Systems Biology Infrastructure).^20^ They try to convince policy makers, funding organizations and industry by paying particular attention to the impacts for human health, for instance the metabolic system (obesity) or personalized medicine (Hood et al. 2012). However, as these applications of systems biology will take considerable time to develop and no immediate results can be promised, industry is reluctant to make big investments and government only provides short-term investments.

In general, the temporary character of investments in large-scale biology distinguishes it from big physics, which is based on long-term funding investments in large instruments. Following the sequencing centers, national investments in large-scale biology have repeatedly taken the form of temporary projects or new centers—for example centers of systems biology and synthetic biology—funded over periods of five years, a time that is more in keeping with the political than the scientific cycle (see also Vermeulen 2015). As there are no large instruments involved, it is much easier to end such investments, thus impacting the continuity of scientific work and the career of young researchers involved. As in some forms of biology, long-term research is a prerequisite to answer questions about long-term developments; scientists face the challenge to find funding that is big in terms of time. The Long Term Ecological Research Network is an example of a distributed large-scale research network that was able to extend over time, but it is an exceptional case (Parker et al. 2010).

### The Internationalisation Obstacle

As already becomes apparent in the attempts to scale-up systems biology, it is difficult to find funding for large-scale international research projects. We are living in a globalized world where—and this is particular true in the scientific realm—internationalization prevails. Yet at the same time large-scale research projects that go beyond national and regional (e.g. European) borders report difficulties in finding funding (Vermeulen 2009). While on the one hand science policy is promoting internationalization by harmonizing higher education, stimulating international mobility of researchers, and emphasizing international publications, it seems to lack the accommodation of large-scale international research projects. This is especially problematic in the field of biology, as the often-dispersed research material requires working on a global scale. How can we understand this internationalization obstacle?

A good example of a project confronted with the problems of international collaboration is the Census of Marine Life (CoML). This large-scale international project dedicated the first decade of the twenty-first century to assessing the diversity, distribution, and abundance of marine life in the past, present and future (Vermeulen 2012). With an emphasis on the present, 2700 marine biologists from 80 countries mapped life in the various spaces of the world’s oceans—from the shore to the deep-sea; from the Antarctic to the Caribbean—storing their research results in the Ocean Biographic Information System (OBIS) that developed over the course of the project. While finding research funding for marine biology research is already extremely difficult, it is almost impossible to find funds for international collaboration. Or as a French member of the scientific steering committee stated: “It is true that if you have a connection with a different country or laboratory, it is not easy to fund it. While it is very worthy to work together, so I think it’s stupid”.^21^ To overcome the funding problem CoML has found the New York based Alfred P. Sloan Foundation prepared to cover the costs of international coordination, while finding research funding from more local sources. With this creative solution CoML first stimulated the cooperation between the United States and Europe. A specialist in *Chaetognatha* (arrow worms) pointed out: “This kind of collaboration has great additional value as people in Europe and the US have different specializations that we can now bring together which gives us new insights”.^22^ And after covering the East and West Atlantic the Census soon spread towards other regions as well: “Many countries, including India and China, have strong research programmes in marine biodiversity, which should enhance the longer term focus on Census related issues” says a steering group member from Canada.^23^ And with the creation of regional and national nodes in Australia, Canada, the Caribbean, China, Europe and the Indian Ocean, the Census has found a localized approach to the internationalization of research.

When comparing the problems of global expansion in current large-scale biology with big physics, it becomes clear that while research has become increasingly internationalized, science policy and especially its funding schemes have not been able to keep up with this trend. Big science is primarily a product of American society and initially did not cross national borders. As the United States is in itself quite big, it has been able to build big physics and large-scale space programs within its national borders during a time when science was used to create national power on an international playing field. However, big biology becomes big in a time when research is very much an international endeavor, while countries are still only prepared to fund national research, and only few scientific funding organizations have agreements to co-fund international research. Through its political, economic and scientific integration the European Union has also acquired a space that allows large-scale international research, but due to its cooperation structures scientific collaborations need to be formed within pre-defined spaces and timeframes, which do not always match with scientific requirements. Moreover, scientific projects funded by the European Commission need, in accordance with European Law, to contribute to innovation.^24^ This excludes fundamental research projects like the Census of Marine Life.

The gap between international orientated big biology and national orientated funding obviously complicates the building of large-scale biology research. The more so as big biology with its distributed network character is more globally spread than the more centralized big physics. Although this network shape can also be turned into an advantage, since nodes of the network can ask for national funding in the country, creating a patchwork of projects that come together at international level, this strategy also has its drawbacks. First, the experience of CoML shows how additional funding for international coordination is the basic prerequisite for such a patchwork approach. In the case of CoML coordination funding came from the Sloan Foundation, but charitable funds are not always available and often only support specific types of research. Secondly, the patchwork approach excludes countries that have no national funds available for the research topic. In the case of CoML this implied that some important areas of marine biodiversity, most notably around Africa and South-America, could not be covered, thus affecting the research results. Finally, and due to all national research agencies having different requirements, the patchwork consists of a variety of projects with different stakeholders, methods and goals, as well as different timing and coverage of space. This diversity makes the harmonization of research difficult or even impossible. While CoML managed to solve this problem, as the database constructed is a quite loose form of research integration that accommodates the inclusion of various forms of research results, in the case of systems biology such a lack of harmonization is problematic when aiming to construct a functioning model of life, which requires a uniform research processes and standards.

### The Innovation Obstacle

Another obstacle in big biology relates to the ways in which expectations of science have changed over time, as reflected in requirements for research funding. The solid organizational machinery of classic forms of big science was built in times of the old social contract, when the strong belief prevailed that basic science would lead to societal progress. In contrast, nowadays science is expected to present a clear concept of the economic and social benefits of its research for the public good in order to get funding (Douglas 2005; Nowotny et al. 2001; Rip 1998). However, while research into life has many potential applications that are useful for society, especially big biology has to prove its innovative potential, with important consequences for the building of large-scale collaboration.

Benefits of big biology are not assumed and life scientists are always expected to know future (industrial) applications of their research. And while they are often able to invoke substantial potential impacts on the improvement of health, nutrition or environment, they also have to address failed promises and ethical issues. As a result system biologists, for instance, not only need to demonstrate the benefits of their own research, but must also explain how their promises will turn into reality, despite the fact that the Human Genome Project did not realize its promise of finding cures for all major diseases. In addition, they need to foresee ethical implications of their research in order to find ways to deal with these issues in a proper way, although these issues are rarely straightforward and often difficult to profess.^25^ Consequently, biology needs to build its bigness within an environment that values application above more basic forms of science, and in order to get funding for research, clear arguments should be in place on societal impacts including ethical implications. Moreover, to guarantee the applicability of research, funding programs often require collaboration with industry.

For instance, the Dutch Genomics Initiative that was in charge of improving genomics research in the Netherlands, decided to fund so-called,innovative research projects’ which require industry to formulate the research question and lead the research consortium (Vermeulen 2015). So when Dr. Arno Andeweg, a virologist from the Erasmus University in Rotterdam, wanted to use genomics to investigate the interaction between host and respiratory viruses (like influenza), he was confronted with these specific criteria to acquire funding. Consequently, he fitted the research plans of his so-called VIRGO project—the acronym being a contraction of virology and genomics—into the funding format. He involved companies with whom his department previously worked together and gave a spin-off company of the department the lead. And as international partners were not allowed, he also kept the consortium strictly national, in spite of virology being a field in which international collaboration is central. These adaptations of the research plan proved to be successful, as the project raised the funds.

Yet, the VIRGO case shows how this requirement for innovation might also distract from performing good research that is applicable. To adapt to funding requirements, the virology research had to align with practices in both government and industry, which influenced scientific practice.^26^ For instance, when mediating between the different time frames of the research process, annual administrative time, and industrial, time to market’ (TTM), the project practice shows that administrative time often got the upper hand:

In the summer [of 2003] we already knew that we were in second position concerning the science review of about seventy projects that would eventually get funded, so we knew we had a very big chance. But it took almost a year before we actually got the letter that we could start. That was around March or April 2004. And to make matters worse, we had to start retro-actively in January.^27^

This also meant that the first evaluation took place just after the project started with the review report stating: “The Committee were really only able to review planned activities and preliminary data. Productivity for all Work Packages was impossible to assess” (Johnston et al. 2007: 11). Moreover, the emphasis of the Netherlands Genomics Initiative on innovation even made them ask the expected amount of patents from the project, neglecting the unpredictable character of research:

If you can predict what will be the result of your research, you do not have to perform the research anymore […] And they want it [the number of patents] specified per year. It is like having to predict in which city you will live 10 years from now and also knowing in which street and at which number […] If I already knew, I would not be working in academia but I would work as an adviser to a company.^28^

As these difficulties of the innovative project VIRGO exemplify, possessing the potential for innovation may also complicate the establishment of large-scale research projects as well as the research practice.

Moreover, the requirement to innovate fuels competition between individuals and groups, which can be an obstacle to collaboration. This became apparent for instance, through a largely empty database within a European Framework project on systems biology. While the sharing of data is a key objective when wanting to model life, the career of post-docs working on the experiments is depending on their ability to publish first-authored papers in good journals, something that can only be done based on sound and exclusive data showing innovation. As such, they are reluctant to share their data, and rightfully so, as their future is hanging in the balance.^29^ In addition, the groups working together in the context of the German Liver Network were at the same time competing against each other for other types of research funds, which obviously also puts a strain on openness and collaborative spirit.

## Conclusion

Miroslav Radman, a successful Croatian life scientists who works in the TAMARA lab in France and has set-up the Mediterranean Institute for Life Sciences in his homecountry, made an analogy between the collective performance of science and music.^30^ While small-scale creative collaborations can be compared to jazz bands, large-scale collaborations can be seen as symphony orchestras. While small jazz bands improvise, and their music is the product of a playful interaction between musicians that are able to inspire each other to perform wonderful solos, the symphony orchestras are much bigger, everybody must be synchronized, knowing exactly what they will play. Although this is a wonderful metaphor for scientific collaboration, it may appear a bit too black-and-white as also in large-scale collaborations there might be room for some improvisation and surprise within the set-up of organizational structures. Therefore, I propose to compare big science with a big band: a large group of carefully arranged people, directed by a band leader, playing pre-set musical arrangements with now and then room for improvisation.

Taking this metaphor of big science as a big band or a symphony orchestra a step further, reveals that in both cases not only the musicians or the scientists are important, but that also the environment in which they act is crucial. The quality of the music does not only depend on the musicians and their instruments, but also on their musical education, the repertoires available, the skills of the conductor, the form of the building in which the music is played, the interest of the audience, and the cultural atmosphere of the country in which the orchestra is based. The same applies to big science. The character of big science is not only determined by the number of scientists involved, the amount of money invested in research, or the size of the instruments—scientific ensembles are also influenced by the subject of research, the questions they ask, and the social and political environment in which they are embedded.

As such, and as I have argued in this chapter, taking a big science perspective, makes it possible to compare the construction of large-scale collaborations in biology to similar movements in other fields, not only comparing different types and structures related to epistemic differences, but also comparing specific forms of historical and cultural situatedness. By emphasizing this interplay between science and its context, the analysis of biology through the lens of big science, also exposes specific difficulties in the interaction between the building of large-scale biology and the contemporary societal context. Consequently, the concept of big science can improve the understanding of the dynamics of large-scale collaboration in biology and other research areas and still be a valuable addition to the contemporary studies of scientific collaboration (e.g. Shrum et al. 2007; Hackett et al. 2016; Cooke and Hilton 2015).

## Figures and Tables

**Table 1:** An overview of differences between big physics and big biology

Centralised big physics	Networked big biology
Centralised organisation	Decentralised organisation
Concentration of research around large and expensive instruments	Networked collaboration underpinned by information infrastructures
Emerged in beginning of 20th century in context of physics	Emerged towards the end of the 20th century in context of molecular biology
Part of modern society characterised by believe in progress, growth, rationalisation, industrialisation and bureaucratisation	Part of contemporary society characterised as post-industrial, knowledge society/economy, neo-liberal capitalism, globalisation.
Science as source of power	Science for economic growth
Industrialisation of science	Embedding of science in society
Social contract between science and society: protected space for (basic) science with continuous government support	New social contract between science and society: emphasis on application, innovation and societal implications of science

## Endnotes

1 Interestingly, most studies on the Human Genome Project focus on the legal, ethical and social implications of the research and not on organisational transformations in research. Dreger (2000) argues that the budget for ELSI research that came together with the Genome project “has tended to keep analysis focused no the potential applications of genetic research, rather than on the nature, meaning, and propriety of the research itself” (ibid.: 171).

2 Interview with Professor Roel van Driel, University of Amsterdam. Amsterdam: March 30, 2005.

3 This study is limited to research based in academia, and has not looked into industrial collaborations, as those types of collaboration are already extensively analyzed elsewhere, especially in literature on public-private collaboration.

4 See also “Tribute to Alvin M. Weinberg”, *Oak Ridge national Laboratory Review* (28), URL: http://www.ornl.gov/info/ornlreview/rev28-1/text/wbg.htm (last accessed 28.03.2006).

5 While the big science concept has a quantitative and a qualitative side, I focus here especially on the latter one. Nevertheless, also when taking a bibliometric view the growth of biology is confirmed through the growth in publications (see Vermeulen et al. 2013).

6 The relation between DoE and NIH with regard to the HGP was discussed during the scientific symposium ’From Double Helix to Human Sequence and Beyond’ at the National Institutes of Health, Bethesda/Washington DC, April 14–15, 2003.

7 The spectrometer has its origins in the physical and chemical sciences but has been appropriated to biology from the 1930s onwards (Zallen 1992). In short, spectroscopy uses light and its absorption pattern to determine substances in a sample and it is used in biology to characterise interactions between molecules.

8 To illustrate, the first NMR apparatus that De Bie bought had a price tag of 150,000 Dutch guilders—which equals less than 75,000 euro—while the last NMR he acquired cost 6 million euros. Although the last machine was probably 10,000 times better then the first “it indicates the enormous increase in scale”. Interview Dr. Rien de Bie, Bijvoet Centre, University of Utrecht. Utrecht: November 10, 2006.

9 The Bijvoet Center is a collaboration between Utrecht University and the Netherlands Foundation for Chemical Research (SON) devoted to structural biology. Information retrieved from the website of the Bijvoet Centre, Historical Background, URL: http://www.bijvoet-center.nl/about/history (last accessed 30.11.2006).

10 Interview with Dr. De Bie (2006).

11 Interview with Professor Dianna Bowles, Biology Department York University, York: August 30, 2006 and Dr. De Bie (2006).

12 Interview with Professor Bowles (2006), interview with Professor Dale Sanders, Head of the Biology Department, York University, December 12, 2005 and interview with Dr. Marianne van der Woude, Biology Department York University, December 15, 2005.

13 Interview with Professor Bowles (2006).

14 According to Pillmoor, the director of the Technology Facility. Pillmore cited in *Bioscience Case Study* on University of York Technology Facility. Retrieved from the web, URL: http://www.bioscience-yorkshire.com/assets/oldassets/bio/c_York_University.pdf (last accessed 03.12.2006).

15 Examples of such databases are, GenBank’ and, Ensembl’. Genbank is managed by the NCBI of the NIH and, Ensembl’ is a cooperation between the European Molecular Biology Laboratorium, the European Bioinformatics Institute, and the Sanger Institute.

16 Site visit CERN and interview Professor Kors Bos, computation coordinator of the ATLAS experiment, CERN, Geneva, Switzerland: April 30, 2008.

17 For the Forward Look on systems biology see URL: http://www.esf.org/index.php?eID=tx_nawsecuredl&u=0&file=fileadmin/be_user/research_areas/emrc/FL/SYSTEMS%20BIOLOGY.pdf&t=1412683083&hash=e397a86e9306ad805e0fa0b9af1cb2faa1726ff8 and see for the European flagship project ITFoM http://www.itfom.eu/.

18 Interview with Professor Bos (2008). See also Kevles (1997) on the death of the SSC.

19 Interview with Dr. Adriano Henney, program director of the Virtual Liver Network, Manchester March 4, 2012. For the Virtual Liver Network see URL: http://www.virtual-liver.de/.

20 For more information on the Neercanne Initiative see URL: http://www.ncsb.nl/workshop/neercanne; and for the European Infrastructure for Systems Biology go to URL: http://project.isbe.eu/.

21 Interview with Professor Myriam Sibuet, IFREMER, France. Maastricht: September 20, 2006.

22 Interview with Dr. Annelies Pierrot-Bults, University of Amsterdam. Amsterdam: January 8, 2007.

23 Interview with Professor Michael Sinclair, Bedford Institute of Oceanography in Canada. Maastricht: September 20, 2006.

24 Interview with Dr. Henriette van Eijl, DG Research, European Commission, Brussels, Belgium, April 19, 2005.

25 This became very apparent in the workshop, New Biology and Society: Opportunity, Challenges and Myths’ at the Lorentz Center of the University of Leiden, January 31–February 3, 2011 (URL: http://www.lorentzcenter.nl/lc/web/2011/431/info.php3?wsid=431).

26 See also Vermeulen (2015) where I have diagnosed this as a process of asymmetrical convergence, in line with the work of Kleinman and Vallas (2001).

27 Interview with Dr. Arno Andeweg, Erasmus Medical Centre, Rotterdam: April 29, 2005 and Utrecht: November 14, 2012.

28 Ibid.

29 These examples are derived from interviews and fieldwork on the emergence of systems biology in Manchester and Berlin in the period 2012–2014.

30 See TAMARAs lab (URL: http://www.necker.fr/tamara/) and Mediterranean Institute of Life Sciences (URL: http://www.medils.org).
